# Health inequity in the Northern Territory, Australia

**DOI:** 10.1186/1475-9276-12-79

**Published:** 2013-09-13

**Authors:** Yuejen Zhao, Jiqiong You, Jo Wright, Steven L Guthridge, Andy H Lee

**Affiliations:** 1 Northern Territory Department of Health, PO Box 40596, Casuarina, NT 0811, Australia; 2 School of Public Health, Curtin University, GPO Box U 1987, Perth, WA 6845, Australia; 3 Health Gains Planning Branch, Department of Health, NT, Australia; 4 Activity Based Funding Branch, Department of Health, NT, Australia

**Keywords:** Indigenous health services, Morbidity, Mortality, Poverty, Socioeconomic factors

## Abstract

**Introduction:**

Understanding health inequity is necessary for addressing the disparities in health outcomes in many populations, including the health gap between Indigenous and non-Indigenous Australians. This report investigates the links between Indigenous health outcomes and socioeconomic disadvantage in the Northern Territory of Australia (NT).

**Methods:**

Data sources include deaths, public hospital admissions between 2005 and 2007, and Socio-Economic Indexes for Areas from the 2006 Census. Age-sex standardisation, standardised rate ratio, concentration index and Poisson regression model are used for statistical analysis.

**Results:**

There was a strong inverse association between socioeconomic status (SES) and both mortality and morbidity rates. Mortality and morbidity rates in the low SES group were approximately twice those in the medium SES group, which were, in turn, 50% higher than those in the high SES group. The gradient was present for most disease categories for both deaths and hospital admissions. Residents in remote and very remote areas experienced higher mortality and hospital morbidity than non-remote areas. Approximately 25-30% of the NT Indigenous health disparity may be explained by socioeconomic disadvantage.

**Conclusions:**

Socioeconomic disadvantage is a shared common denominator for the main causes of deaths and principal diagnoses of hospitalisations for the NT population. Closing the gap in health outcomes between Indigenous and non-Indigenous populations will require improving the socioeconomic conditions of Indigenous Australians.

## Introduction

The past several decades have seen substantial improvements in the average level of health measured by mortality rates in many countries [1]. These trends are evident for both developed and developing countries. However, health inequalities have remained static or widened around the globe [2]. In Australia, there is a wide gap in life expectancy at birth between Indigenous and non-Indigenous populations [3]. It has been suggested that Australia was the only country in which the gap was large and widening, compared with similar developed countries with significant Indigenous populations [4]. The disparity in health outcomes between two subpopulations in Australia has been acknowledged by health authorities as a major social and public health challenge. Australian governments have declared their intentions to close this life expectancy gap within a generation, and to halve similar gaps in education and employment within a decade [5].

The Northern Territory (NT) is situated in northern and central Australia, and has the smallest population (231,331 in 2011) among all federal states and territories. The NT covers about one sixth of the landmass of Australia, but comprises only 1% of the total Australian population. It has higher proportions of people in remote and very remote areas than any other state or territory. Aboriginal and Torres Strait Islander (hereafter referred to as Indigenous) people constituted 30% of the total NT population and experienced poor health status [3]. In 2006, the NT Indigenous life expectancy at birth was 60 and 70 years for males and females respectively, with corresponding gaps of 21 and 15 years between Indigenous and non-Indigenous populations [6]. The majority of the Indigenous population (70%) live in remote and very remote areas, and almost 75% of the Indigenous population live in poor areas. It remains uncertain how to overcome the persisting health inequalities and to what extent socioeconomic determinants contribute to that difference [7].

Socioeconomic status (SES) refers to an individual or family’s economic and social position measured by income, education and occupation [8]. Socioeconomic inequalities in health describe the variations in health status between different socioeconomic groups of the population [9,10]. There is a growing body of literature highlighting socioeconomic inequality in health in Australia and internationally [7,11-13]. The poor tend to die earlier and have higher levels of morbidity than the better-off. Low SES affects health adversely throughout the life course and between generations [14,15]. Evidence suggests that causality between poverty and poor health runs in two directions: poverty generates ill-health, ill-health exacerbates poverty [9]. A further factor is that there is a strong connection between poor health, poverty and remoteness [16-18]. Earlier studies in the NT reveal a clear social gradient to the prevalence of diabetes and burden of disease measured in disability adjusted life years (DALY) [19,20]. However, there have been no previous studies on the contribution of socioeconomic disadvantage to Indigenous health disparities and the links between health outcomes, socioeconomic determinants, Indigeneity and remoteness.

In this study, we aim to describe health inequalities in mortality and morbidity for the NT population and to examine the extent to which the Indigenous health gap may be attributed to differences in SES. An understanding of the socioeconomic impact on health is of central importance in informing strategies to address the Australian governments’ commitment to closing the Indigenous health gap [5].

## Methods

This is an observational study on SES related mortality and morbidity outcomes for the total NT population using three main data sources gathered from 2005 to 2007: deaths, public hospital admissions and estimated resident population [3]. The study period was chosen because of data availability with the 2006 Census data available for the middle year of the study. Sourced from the Census, the Socio-Economic Indexes for Areas (SEIFA) were designed to measure SES for a geographic area in Australia [21]. The Accessibility/Remoteness Index of Australia (ARIA) was developed by the University of Adelaide, based on road distances to population centres for the purposes of measuring remoteness and accessibility to goods and services. For more information about ARIA classification, see Reference [22]. Deaths, hospital admissions, burden of disease, and population data were linked by age group, sex, Indigenous status and Statistical Local Areas (SLA) of residence, while SEIFA and ARIA scores were available at the SLA level.

SEIFA is a system of national rankings of neighbourhood SES for geographic areas, derived from over 30 socioeconomic variables collected in the Census [21]. These variables measure the socioeconomic advantage and disadvantage in multiple dimensions including income, education, employment, occupation and housing. There were four types of SEIFA scores: index of relative socio-economic disadvantage, index of relative socio-economic advantage and disadvantage (IRSAD), index of economic resources and index of education and occupation [21]. For all four indices, a higher SEIFA score indicates better SES for the area, and vice versa. In this study, we used IRSAD as a measure of socioeconomic inequality to avoid the collinearity between SES and Indigenous status, because IRSAD is the only index which does not contain Indigenous proportion in its composition. Refer to the 2006 SEIFA website and explanatory notes for more information [23]. SLAs were grouped into three area-based socioeconomic categories: low (IRSAD < 958), medium (958–1,043) and high (>1,043). Quintiles were used to estimate rate ratios. The IRSAD does not include remoteness measures. SLA is a general purpose spatial unit used for statistical reporting by the Australian Bureau of Statistics [24]. The 2006 SEIFA included 90 SLAs in the NT. The median SLA population size in 2006 was 1,812 (range 88–14,323 persons) and the median area size was 12.9 square kilometres (range 0.3–195,402) in the NT. The SLAs were classified into three remoteness categories, using ARIA scores: non-remote (0–5.79), remote (5.80–9.07) and very remote (9.08–12) [22]. The 1999-2004 DALY estimates were used to project the burden of disease estimates for 2006, based on the observed demographic changes. DALY is a composite measure of premature mortality and disability measured in healthy years of life lost [25]. Premature deaths were calculated using the standard life expectancy at the age of death. Disability was estimated by an agreed disability weights [25]. The DALY data were further analysed by region and SLA to provide health information for regional planning. The total burden of disease was estimated using DALY rates. The regions are Darwin Urban, Darwin Rural, Katherine, East Arnhem, Alice Springs Urban, Alice Springs Rural and Barkly.

Death registrations between 1 January 2005 and 31 December 2007 were obtained from the Australian Bureau of Statistics, and were based on deaths recorded by the Registries of Births, Deaths and Marriages. Causes of death were coded in the International Classification of Diseases and Related Health Problems, 10th Revision. Age at death, sex, Indigenous status and residential SLA were also available from the death data. The underlying causes of death were analysed by chapter (the first digit of the code), which includes infectious disease, cancer, metabolic/nutritional disease, mental disorder, diseases of the circulatory, respiratory, digestive, musculoskeletal and genitourinary systems, conditions originating in the prenatal period, and injuries. We used the number of deaths per 1000 population to show mortality inequality.

NT public hospital admissions from 2005 to 2007 were used to measure morbidity. The hospital morbidity was measured by hospitalisations per 1000 population. There are five public hospitals in the NT: Royal Darwin Hospital, Alice Springs Hospital, Katherine Hospital, Gove District Hospital and Tennant Creek Hospital. In Australia, hospital services are provided free of charge to all public patients. Hospital morbidity reflects the acute care needs of the general population. Patient demographic information (age, sex, Indigenous status and residential SLA) was recorded during each admission. The principal diagnosis was analysed by the chapter codes of the International Classification of Diseases.

Because the focus of this study was on SES related health inequality, we used age-sex standardisation to improve comparability between different groups, as suggested in Reference [26]. The standardisation method is based on the concept of horizontal equity, which assumed that the health status for people of the same age and sex should be comparable. All health measures were adjusted to the standard Australian 2006 estimated resident population by both age group and sex using direct standardisation [27]. The Gini index and the concentration index were used to describe socioeconomic and Indigenous inequality in health, as recommended by the World Health Organisation [9,28,29]. The Gini index measures the total health inequality with the minimum being 0 and maximum 1. Concentration index measures the socioeconomic related inequality in health with the minimum −1, maximum 1, and 0 indicating there is no socioeconomic health inequality. A negative concentration index demonstrates that a selected measure is a protective factor and vice versa. The Gini index, concentration index and their decomposition techniques allow the assessment of the socioeconomic impact on health [30]. Multivariate analysis is well suited for controlling confounders and correcting bias [31]. Poisson regression was used to analyse age group, sex, Indigenous status, SES and remoteness simultaneously [32]. Multivariate analyses were performed using Stata/IC 12.0.

## Results

The mortality and morbidity data are compared with population information in Table 1. Between 1 January 2005 and 31 December 2007, 2,815 deaths and 253,017 hospital admissions were recorded for NT residents. Of these, 46% deaths and 68% hospitalisations were for Indigenous people, both substantially greater than the proportion of Indigenous people in the total NT population (30%). A gender difference was also evident: 63% of deaths and 57% of hospitalisations were recorded for males, clearly higher than the proportion of males in the total population (52%). There were also marked differences in both deaths and hospitalisations between age groups. As a result of these differences it was necessary to perform age-sex standardisation when analysing Indigenous health inequality.

**Table 1:** Deaths and hospital admissions by sex and Indigeneity, Northern Territory, Australia, 2005-2007

	**Deaths**		**Hospital admissions**		**Population in 2006**	
	**Male**	**Female**	**Male**	**Female**	**Male**	**Female**
**Indigenous**	747	561	102639	68615	31499	32509
**Non-indigenous**	1027	480	42667	39096	77393	69237

**Figure 1:**
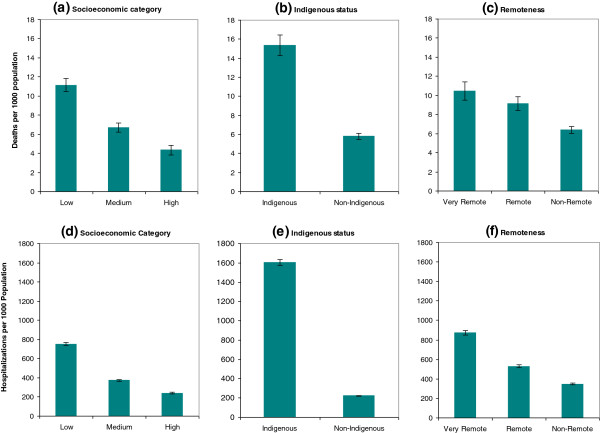
**Health inequalities in mortality and hospital morbidity by socioeconomic factors with 95% confidence intervals, Northern Territory, Australia, 2005–2007.** Legend: **(a**, **b**, **c)** Age-sex standardised mortality rates by area-based socioeconomic category **(a)**, Indigenous status **(b)** and remoteness category **(c)**. **(d**, **e**, **f)** hospital morbidity rates by area-based socioeconomic category **(d)**, Indigenous status **(e)** and remoteness category **(f)**.

**Figure 2:**
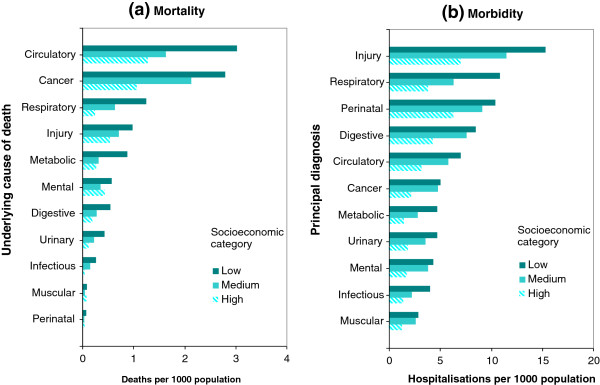
**Health inequalities by socioeconomic categories and conditions, Northern Territory, Australia, 2005–2007.** Legend: **(a)** Age-sex standardised mortality rates by socioeconomic categories and underlying cause of death; **(b)** Hospitalisation rates by socioeconomic categories and principal diagnosis.

By comparing the three different socioeconomic groups (see Figure 1(a)), the age-sex standardised mortality rate for the low SES group was almost twice that of the medium SES group, and more than 2.5 times the rate for the high SES group (P < 0.01). In Figure 1(b), the age-sex standardised mortality in Indigenous population was more than 2.5 times greater than their non-Indigenous counterpart (P < 0.01). Figure 1(c) suggests that remoteness was likely to be another contributing factor to inequalities in mortality outcomes (P < 0.05). Age-sex standardised mortality rates were then analysed by the ICD-10 chapter for underlying cause of deaths (Figure 2(a)). The three leading causes of death were circulatory disease, cancer and respiratory disease. A clear SES gradient was found for all disease categories, with the exception of mental and musculoskeletal illnesses. Deaths due to mental and musculoskeletal illnesses were higher in both low and high SES population groups.

**Figure 3:**
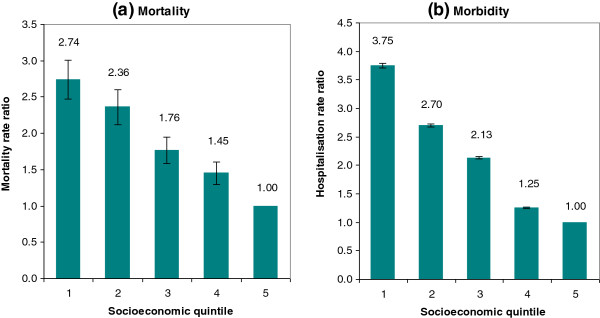
**Rate ratios by socioeconomic quintiles with 95% confidence intervals.** Legend: **(a)** Mortality rate ratio; **(b)** Hospitalisation rate ratio.

Table 2 summarises the total and SES related health inequality measured by Gini index and concentration index, and the contribution of IRSAD and Indigenous proportions estimated by decomposition. The total mortality inequality was 0.33. The concentration indices for IRSAD scores and Indigenous proportions were −0.192 and 0.193 respectively, indicating the mortality was more concentrated among the poor, and the areas with higher Indigenous proportion. The contributions of IRSAD and Indigenous proportion to the total mortality inequality were 28% and 30%, suggesting over one-quarter of mortality inequality is due to socioeconomic factors. Figure 3(a) shows that the socioeconomic disadvantage was linearly associated with increased risk in mortality. The mortality risk in the lowest quintile (most disadvantaged) was 2.74 times that of the highest quintile (least disadvantaged) (P < 0.01).

**Table 2:** Gini index, concentration index and decomposition of health inequality with respect to IRSAD and Indigeneity

	**Gini index**	**Concentration index**		**Decomposition**		**Contribution (%)**	
		**IRSAD**	**Indigeneity**	**IRSAD**	**Indigeneity**	**IRSAD**	**Indigeneity**
**Mortality**	0.330	−0.192	0.193	0.093	0.098	28.0	29.6
**Morbidity**	0.471	−0.264	0.258	0.114	0.122	24.2	25.9

*IRSAD* Index of relative socio-economic advantage and disadvantage.

Hospitalisation rates were also analysed and the results compared with mortality. An overall analysis in Figure 1(d) indicates that people from low SES residential areas were two times more likely to be hospitalised than people from medium SES areas, who were in turn about 50% more likely to be hospitalised than people from high SES areas (P < 0.01). To see the difference in health inequality by Indigenous status, Figure 1(e) shows that Indigenous people were seven times more likely to be hospitalised than non-Indigenous people (P < 0.01). Figure 1(f) reveals that residents from remote areas were 50% more likely to be hospitalised than from non-remote areas, and in turn residents in very remote areas were 60% more likely to be hospitalised than in remote areas (P < 0.01). Figure 2(b) provides more details of age-sex standardised hospitalisation rates by SES category and principal diagnosis. The leading principal diagnosis for hospital admission was injury, irrespective of SES categories. The second leading diagnosis was respiratory disease for the low SES category, and perinatal conditions for the medium and high SES groups. All principal diagnoses of hospitalisations showed consistently that higher hospitalisation rates were associated with low SES and vice versa. This socioeconomic gradient was more marked in injuries, respiratory diseases, perinatal conditions, digestive, and circulatory diseases. For cancer and mental conditions, hospitalisation rates in the low and medium SES areas were similar, two times those in high SES groups.

The total inequality in hospital morbidity was 0.471 (Table 2), slightly greater than that of mortality. The concentration index estimates imply that the hospital morbidity affected the poor, and the Indigenous population disproportionately. The decomposition analysis shows that approximately 25% of the morbidity inequality was associated with socioeconomic factors. Figure 3(b) indicates that socioeconomic status was inversely associated with increased risks in hospitalisation. The hospitalisation risk in the lowest quintile was 3.75 times that of the highest quintile (P < 0.01).

The results in Table 3 confirm that univariate and multivariate analyses are similar. Increasing age and being a male are both associated with increased mortality and morbidity. After adjusting for age group and sex, Indigenous people were 3 times more likely to die prematurely and 11 times more likely to be hospitalised. Improved SES could reduce mortality by up to 50% and reduce hospitalisations by 40% (P < 0.05). Remoteness was associated with increased hospitalisation (P < 0.05) but not mortality (P > 0.05).

**Table 3:** Poisson regression estimates of risk ratios and 95% confidence intervals associated with mortality and morbidity, by key demographics, socioeconomic status and remoteness

		**Mortality**		**Morbidity**
	**Risk ratio**	**95% Confidence interval**	**Risk ratio**	**95% Confidence interval**
**Age group**				
20-39	2.43	1.46-4.01	2.19	2.16-2.22
40-59	9.18	5.8-14.51	5.07	5-5.12
60+	55.26	35.3-86.49	8.57	8.45-8.69
**Female**	0.75	0.56-0.99	0.70	0.67-0.71
**Indigenous**	3.31	0.74-14.67	10.91	10.54-11.29
**Socioeconomic status**				
Medium	0.77	0.68-0.87	0.98	0.96-0.99
High	0.49	0.42-0.57	0.59	0.57-0.6
**Remoteness**				
Remote	0.96	0.38-2.38	1.10	1.08-1.11
Very remote	0.90	0.21-3.87	1.14	1.11-1.17

The Barkly and Alice Springs Rural had the highest burden of disease among the seven regions. The age-sex standardised DALY rates were 2.3 and 1.9 times the NT average respectively (Table 4). Whilst East Arnhem had the lowest DALY rate for the Indigenous and non-Indigenous population, Darwin Urban had the lowest DALY overall. The geographic distribution of ill health is important information for health care policy and service delivery.

**Table 4:** Age-sex standardised DALY rates and rate ratios by Indigeneity and regions, Northern Territory, Australia, 2006

	**Indigenous**		**Non-indigenous**		**Overall**	
	**DALY rate**	**Ratio**	**DALY rate**	**Ratio**	**DALY rate**	**Ratio**
**Darwin Urban**	478.4	1.0	155.1	1.0	180.2	0.8
**Darwin Rural**	364.5	0.8	127.5	0.8	243.6	1.1
**Alice Springs Urban**	614.2	1.3	173.2	1.1	242.5	1.0
**East Arnhem**	338.1	0.7	104.3	0.7	224.6	1.0
**Katherine**	488.1	1.0	181.2	1.2	311.2	1.3
**Barkly**	782.0	1.6	243.6	1.6	532.9	2.3
**Alice Springs Rural**	553.1	1.1	156.9	1.0	432.8	1.9
**Northern Territory**	482.5	1.0	156.7	1.0	231.2	1.0

*DALY* Disability adjusted life year. Rate = DALYs per 1000 population. Rate ratio based on the Northern Territory average.

## Discussion

SES is an important contributor to Indigenous health inequality. Closing the gap between Indigenous and non-Indigenous health outcomes will require both direct health interventions as well as activities that address poverty and social disadvantage. The NT has the highest proportion of Indigenous people and the greatest Indigenous health disparity of all Australian states and territories [33]. This study highlights a clear socioeconomic gradient with both higher mortality and morbidity tied to lower SES. We estimate that approximately 25-30% of the total health disparity for the NT Indigenous population can be attributed to SES. There is an extensive literature on the association between socioeconomic inequality and health [8,9,13,19]. The results from this study are in line with both the majority of international studies, and previous NT studies on burden of disease [20] and type 2 diabetes [19], [34]. However, to our knowledge, this is the first study that has used both mortality and morbidity data to quantify the relative contribution of socioeconomic disadvantage to Indigenous health inequity.

Health inequity is an important social justice issue. Indigenous people have experienced the negative effects of colonisation with many suffering low income, poor education, unemployment, overcrowded accommodation, inadequate sanitation and a lack of essential services [13]. This study shows a close correlation between socioeconomic health inequality and Indigenous health inequality. While it is difficult to ascertain causality, it is intuitive to believe the causality is likely to occur in both directions: poverty causes ill-health resulting from poor nutrition, infectious and chronic disease; and ill-health leads to poverty due to loss of productivity, quantity and quality of life. Attempts to modify risk behaviours, such as smoking, alcohol and obesity, without altering socioeconomic disadvantage will have limited success, because the risk behaviours are often embedded within disadvantage, which in turn reinforces the risk behaviours.

Health inequality can be assessed in many ways. A typical assessment is through describing the differences in health measures and outcomes between different population groups, for example, differences in life expectancy, mortality rate and hospitalisation rate. Another way to assess health inequality is by examining the distribution of health outcomes in the population through use of the Gini index, concentration index, and their refinements and decompositions. These methods allow one to investigate the contributions of determinants to health inequality. Multivariate regression models are helpful in analysing multiple factors simultaneously.

Several limitations need to be considered when interpreting the findings of this report. Firstly, mortality and morbidity data were analysed for only three years, 2005–2007, due to the limited availability of mortality and SEIFA data. We did not have access to the mortality data beyond 2008 as the Australian Bureau of Statistics has ceased all data release while it reviews its policy on use of death records for research purposes. Secondly, the SEIFA scores were area-based SES indices, rather than individual level indices. Previous studies have shown that SEIFA may understate Indigenous disadvantage [35]. As we reported elsewhere [36], a multilevel regression model may be useful in reducing ecological bias. A third limitation is that the Indigenous population estimates were experimental, although the NT estimates had the smallest relative standard error among all Australian jurisdictions [37]. Additionally, public hospital admission is only an approximate measure for the true morbidity, and should be interpreted with care. Private hospital data was not available for this study. There is only one private hospital in the NT (Darwin Private Hospital). The public hospitals provide most of acute care services [38]. Finally, due to a lack of individual level data, this study did not explore SES inequalities within the Indigenous population. The interactions between different axes of health inequality by gender, social class and race have not been explicitly and fully considered in this study. Health inequality by gender was controlled by age-sex standardisation. To address some of these limitations, a future study may consider using linked unit level data. For brevity, only IRSAD results were presented in this paper. The other three SEIFA scores were also analysed and the results were consistent with IRSAD.

## Conclusions

Socioeconomic disadvantage is a shared common denominator for the leading causes of deaths and principal diagnoses of hospitalisations for the NT population. This study linked Indigenous health inequality with socioeconomic inequality, using both mortality and morbidity data. The socioeconomic and geographical analyses in the study will inform interventions that address socioeconomic disadvantage as a mechanism to close the Indigenous health gap. This study has also contributed knowledge and baseline information for future studies on health inequity.

## Abbreviations

ARIA: Accessibility/remoteness index of Australia; DALY: Disability adjusted life years; IRSAD: Index of relative socio-economic advantage and disadvantage; NT: The Northern Territory; SEIFA: Socio-economic indexes for areas; SES: Socioeconomic status; SLA: Statistical local areas.

## Competing interests

The authors declare that they have no competing interests.

## Authors’ contributions

YZ and SLG initiated and designed the study. YZ and JY contributed to data collection and performed the statistical analysis. AHL and JW provided technical support and led the drafting and revision of the paper. All authors read and approved the final manuscript.
